# Elevated water temperatures enhance 2,4,6-trinitrotoluene induced stress response of carbonyl reductase in blue mussels (*Mytilus edulis*): Another environmental impact of climate change?

**DOI:** 10.1007/s00204-026-04520-x

**Published:** 2026-08-25

**Authors:** Jacqueline Lindemeyer, Romina Marietta Schuster, Lili Hartmann, Lillian Tabea Hannah Bünning, Matthias Brenner, Edmund Maser, Jennifer Susanne Strehse

**Affiliations:** 1 Institute of Toxicology and Pharmacology for Natural Scientists, University Medical School Schleswig-Holstein, Brunswiker Str. 10, 24105 Kiel, Germany; 2 Alfred Wegener Institute Helmholtz Centre for Polar and Marine Research, Am Handelshafen 12, 27570 Bremerhaven, Germany

**Keywords:** Submerged munitions, 2,4,6-Trinitrotoluene (TNT), Climate change, Marine ecotoxicity, Carbonyl reductase, Oxidative stress

## Abstract

Toxic explosives leaking from submerged munitions appear as an emerging marine pollutant in recent years. The nitroaromatic compound 2,4,6-trinitrotoluene (TNT) is of particular interest in this context. TNT poses a threat to marine environments and organisms. Previous studies showed that TNT and its metabolites are incorporated in mussel tissue thereby causing adverse effects through cellular oxidative stress. Recently, the impact of TNT on mRNA expression of the enzyme carbonyl reductase (CR) in blue mussels was discovered. This enzyme plays a key part in the cellular antioxidative system. A factor that should be taken into account is the increase in water temperature caused by anthropogenic climate change. To investigate potential synergistic effects of TNT in combination with elevated water temperatures on *CR* expression, blue mussels were exposed to concentrations of 0.1 µg/L, 50 µg/L, and 500 µg/L TNT and water temperatures of 11 °C, 15 °C and 18 °C, respectively, in the present study. Semiquantitative PCR analysis of *CR* mRNA expression in different tissues confirmed that TNT leads to increased mRNA expression levels, especially in gill tissue even at lower concentrations than tested in previous studies. Furthermore, varying water temperatures apparently influenced *CR* gene expression. Our study suggests a higher vulnerability to xenobiotic-induced oxidative stress in case of simultaneous presence of pollution from munitions and elevated water temperature. Our findings also underline the importance of molecular biomarkers like *CR* as a method for assessment of the impact of environmental stressors on the marine ecosphere.

## Introduction

Aside from pollutants such as oil components, microplastics and toxic metals, chemicals leaking from dumped and submerged munitions are an additional factor that puts stress on marine ecosystems worldwide. In the German coastal waters of the North- and Baltic Sea, approximately 1.6 million metric tons of munitions dumped after World Wars I and II pose a threat to the marine ecosphere (Böttcher et al. 2011; Bach et al. 2023). Additional to the hazard of explosion, the environment is at risk due to corroding shells and, thus, subsequently released energetic compounds (EC) from the explosives of munition objects.

Particularly nitroaromatic compounds such as 2,4,6-trinitrotoluene (TNT) and its main metabolites 2-amino-4,6-dinitrotoluene (2-ADNT) and 4-amino-2,6-dinitrotoluene (4-ADNT) came into focus of research in the last years. The metabolites are formed during biotic processes by reduction of a nitro group to an amino group (Juhasz and Naidu 2007). While TNT and its main metabolites can lead to adverse health effects such as cataract, anaemia, and cancer in humans (Bolt et al. 2006), TNT also holds the potential to harm marine organisms such as fish, shrimp, copepods, amphipods, and bivalves (Nipper et al. 2001; Ek et al. 2006; Rosen and Lotufo 2007a; Lotufo et al. 2016; Schuster et al. 2026). Studies in recent years showed that EC can be detected in different marine organisms (Rosen and Lotufo 2007b; Porter et al. 2011; Beck et al. 2022), which once again underlines the significance of TNT as an emerging pollutant in marine ecosystems.

One of the marine organisms that plays a crucial role in these ecosystems is the blue mussel (*Mytilus* spp.). The main types in the *Mytilus* complex include *M. edulis*, *M. galloprovincialis*, and *M. trossulus*. Blue mussels are ubiquitous sessile organisms which are very common in German coastal waters (Millat et al. 2012). Since mussels feed by constantly filtering the surrounding water for nutrients, they are a suitable organism for biomonitoring (Goldberg 1975; Strehse and Maser 2020). In previous studies blue mussels were successfully established as bioindicators for monitoring EC leaking from corroded munitions by actively deploying them at potential pollution hotspots, munitions dumping sites and World War ship wrecks followed by ultra-sensitive GC–MS/MS and LC–MS/MS analyses (Strehse et al. 2017; Appel et al. 2018; Greinert et al. 2019; Maser and Strehse 2020; Maser et al. 2023a, b).

Beyond this chemical analysis approach, research showed that TNT exposition also causes multilevel adverse effects on behavioural and physiological levels in blue mussels (Schuster et al. 2021). Furthermore, gene expression of carbonyl reductase (*CR*) was shown to be influenced by TNT (Strehse et al. 2020). The oxidoreductase CR—belonging to the protein superfamily of short-chain dehydrogenases/reductases (SDR)—is expressed in a wide range of tissues of all phyla—from bacteria to mammals—and plays an important role in detoxification of reactive carbonyl compounds that can be formed by reactive oxygen species (ROS) (Hoffmann and Maser 2007). *CR* gene expression is inducible by the presence of ROS-promoting substances (Ebert et al. 2018; Strehse et al. 2020). Since it is known that TNT and its metabolites cause adverse effects by inducing oxidative stress and formation of ROS in biota during xenobiotic metabolism (Adomako-Bonsu et al. 2024), the analysis of the relative gene expression could successfully be established as a molecular biomarker for environmental TNT contamination in blue mussels (Strehse et al. 2020). Here, the correlation between TNT concentration and *CR* gene expression was proven both in a laboratory exposure experiment and a field study (Strehse et al. 2020). A similar effect could also be shown in *Daphnia magna*, an important model organism for toxicological studies in limnic ecosystems. Laboratory exposure studies showed that gene expression of *CR* in daphnids is inducible by TNT (Jacobsen et al. 2022).

This study aims to investigate whether different TNT concentrations are able to influence *CR* gene expression levels in a temperature depending manner. Against the backdrop of advancing climate change that leads to rising water temperatures, the goal was to gain insights into a possible temperature dependence of *CR* expression. Thus, our study combined these two stress factors by exposing blue mussels to different TNT concentrations at three different water temperatures to determine the effects on *CR* gene expression.

## Material and methods

### Laboratory exposure experiment

For the laboratory exposure experiment, blue mussels (*M. edulis*) were collected off the coast of Sylt on March 18, 2024, and transported to the Alfred Wegener Institute, Helmholtz Centre for Polar and Marine Research (AWI) in Bremerhaven on the same day. Upon arrival, the mussels were immediately transferred into 25 L aquaria filled with natural seawater. After five days, the mussels were gradually acclimated to artificial seawater (30 g/L Coral Pro Salt; Red Sea, Düsseldorf, Germany). Simultaneously, a temperature acclimatisation was conducted to reach the target experimental temperatures of 11 °C, 15 °C, and 18 °C.

**Fig. 1:**
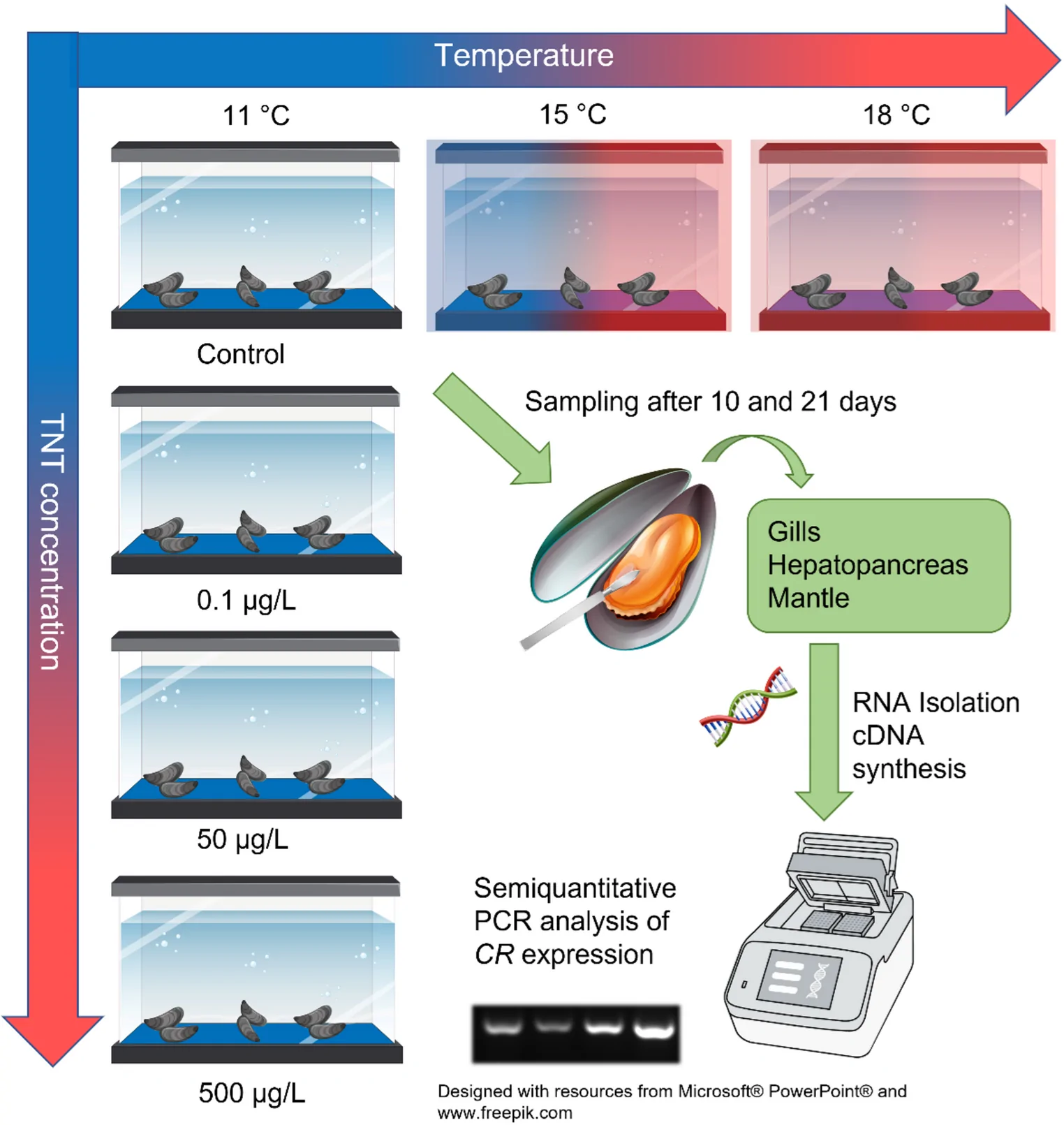
Schematic illustration of the exposure experiment and downstream sample processing. Mussels were exposed to different TNT concentrations (three aquaria (triplicates) per combination of TNT concentration and temperature) at different temperatures for 21 days at the Alfred Wegener Institute. Tissue samples for semiquantitative PCR analysis were taken both after 10 and 21 days

Following a 21-day acclimatisation period, the mussels were transferred into experimental aquaria with a volume of 12 L (three aquaria per treatment, 36 in total). These aquaria were filled with artificial sea water and TNT solutions at target concentrations of 0.1 µg/L, 50 µg/L, and 500 µg/L, along with a control group without TNT (Fig. 1). The TNT solutions were freshly prepared each day by adding a stock solution (100 mg/L in demineralised water; GEKA, Munster, Germany). A complete water exchange was performed daily, in which aquaria were fully emptied and refilled with freshly prepared artificial seawater containing the corresponding TNT concentrations. Each tank was stocked with 12 mussels.

Throughout the acclimatisation and exposure phases, a light:dark cycle of 8:16 h was maintained. Mussels were fed every three days with PhytoMaxx (Nyos Aquatics GmbH, Korntal-Münchingen, Germany), always 1-h prior to the daily water change. The pump was switched off during feeding time.

For sampling, one mussel per temperature group was taken before starting the exposition (baseline samples) and one mussel per aquarium was sampled at 10 and 21 days after start of the exposition period.

### Tissue isolation, RNA extraction and cDNA synthesis

For gene expression analysis, mussels were treated following a protocol described by Strehse et al. (2020). Mussel shells were opened and a maximum of 20 mg of gill, hepatopancreas and mantle tissue was transferred into 1.5 mL nuclease-free reaction tubes, 200 µL of RNA*later* solution (Thermo Fisher Scientific, Waltham, MA, USA) were added to preserve RNA, samples were incubated overnight at 4 °C and kept at -20 °C until further processing. Tissue samples were transferred into lysis buffer from the commercially available Roti^®^-Prep RNA MINI kit (Carl Roth, Karlsruhe, Germany) and homogenised using a T25 Ultra-Turrax (Ika Works Inc., Staufen im Breisgau, Germany). RNA was isolated according to the manufacturer’s protocol and quantified using a Qubit^®^ 2.0 Fluorometer (Life Technologies, Carlsbad, CA, USA) and the Qubit^®^ HS RNA assay kit (Life Technologies, Carlsbad, CA, USA). The isolated RNA was flash frozen in liquid nitrogen and stored at − 80 °C until further processing. An amount of 500 ng RNA was reverse transcribed using RevertAid H minus reverse transcriptase (Thermo Fisher Scientific, Waltham, MA, USA) and 200 ng of Random Hexamer Primers (Promega, Madison, WI, USA). Incubation was performed at 25 °C for 10 min, followed by 60 min at 42 °C and 10 min at 70 °C. Obtained cDNA was stored at -20 °C.

### Gene expression analysis by semiquantitative PCR

Semiquantitative PCR for gene expression analysis was performed using Phire Green Hot Start II DNA polymerase (Thermo Fisher Scientific, Waltham, MA, USA) with 1.5 µl cDNA, 20 pmol primers and 200 µM dNTPs (Life Technologies, Carlsbad, CA, USA) in a total volume of 20 µL. For *CR* and *18S rRNA*, which was used as housekeeping gene, primers published Strehse et al. (2020) were used. Thermo cycling was carried out with initial denaturation at 98 °C for 30 s and following amplification cycles (6 for *18S rRNA* and 24 for *CR*, respectively) consisting of 5 s at 98 °C, 5 s at 50 °C and 15 s at 72 °C. A final elongation step was performed at 72 °C for 1 min. A volume of 18 µL of PCR product was subjected to gel electrophoresis on 1% agarose gels containing GelRed™ (Biotrend, Cologne, Germany) for visualisation. For digital documentation, an Intas^®^ Gel-imaging system (Intas, Göttingen, Germany) was used and gel bands were densitometrically analysed with GIMP 2.10.38 software. The *CR/18S rRNA* ratio was calculated for each sample for semiquantitative gene expression analysis.

### Chemical analysis of EC in mussel tissue and water

Samples for chemical analysis were stored at -20 °C until workup. Water from the aquaria and mussel samples were prepared with a solid phase extraction method for chemical analysis following the protocol described by Bünning et al. (2021) using an amount of 190—500 mg freeze-dried mussel tissue and 1 mL aquarium water (nominal concentration 50 and 500 µL/L) or 50 mL (nominal concentration 0.1 µg/L and negative control) per sample. Samples were analysed with a Thermo Scientific TSQ 8000 EVO GC–MS/MS system on Thermo Scientific TG-5MS amine columns (15 m × 0.25 mm × 0.25 µm), as described by Bünning et al. (2021). The quantification was carried out using external calibration with standards of TNT, 4-ADNT and 2-ADNT.

### Data analysis and statistic evaluation

Mean values of the concentration of TNT and its metabolites 2- and 4-ADNT in mussels and water are to be understood as mean values ± standard deviation based on three technical replicates. Descriptive statistics of the densitometric results obtained from semiquantitative PCR analysis were calculated with GraphPad Prism (Version 10.3.1). Control and exposure groups were analysed for differences using a two-way ANOVA followed by a Dunnett’s test. For groups not following a normal distribution, a logarithmic transformation was applied beforehand. Some samples of the mantle tissue could not be statistically analysed due to a lack of replicates.

## Results

### *CR* mRNA expression

**Fig. 2:**
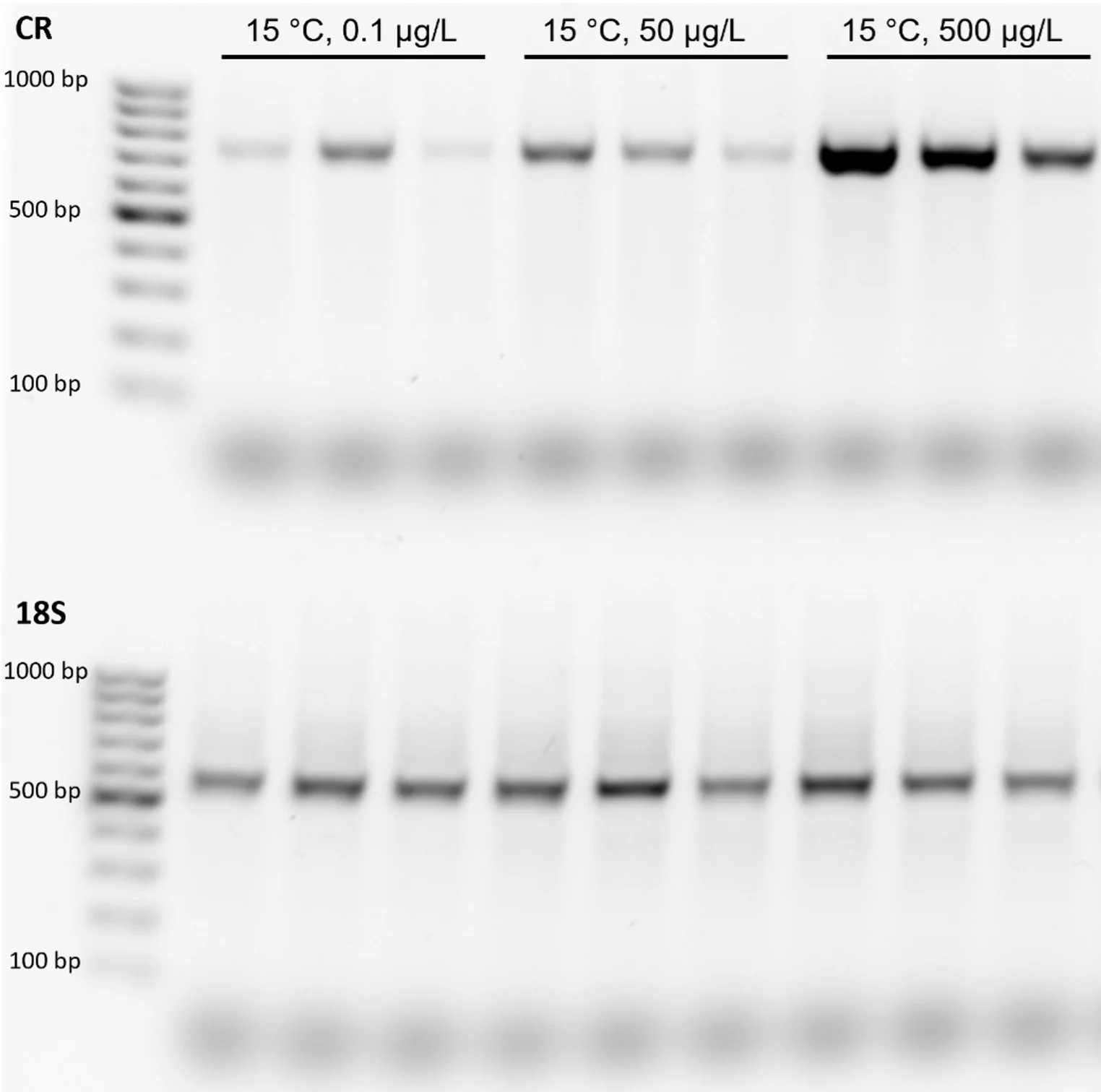
Exemplary gel electrophoresis of samples from the gills of blue mussels exposed to different concentrations of TNT at different temperatures. Samples were exposed during a laboratory experiment at the Alfred Wegener Institute and examined for the expression of *CR* mRNA by semiquantitative PCR analysis. The *18S rRNA* gene was used as housekeeping gene. All bands shown are from the same gel

**Fig. 3:**
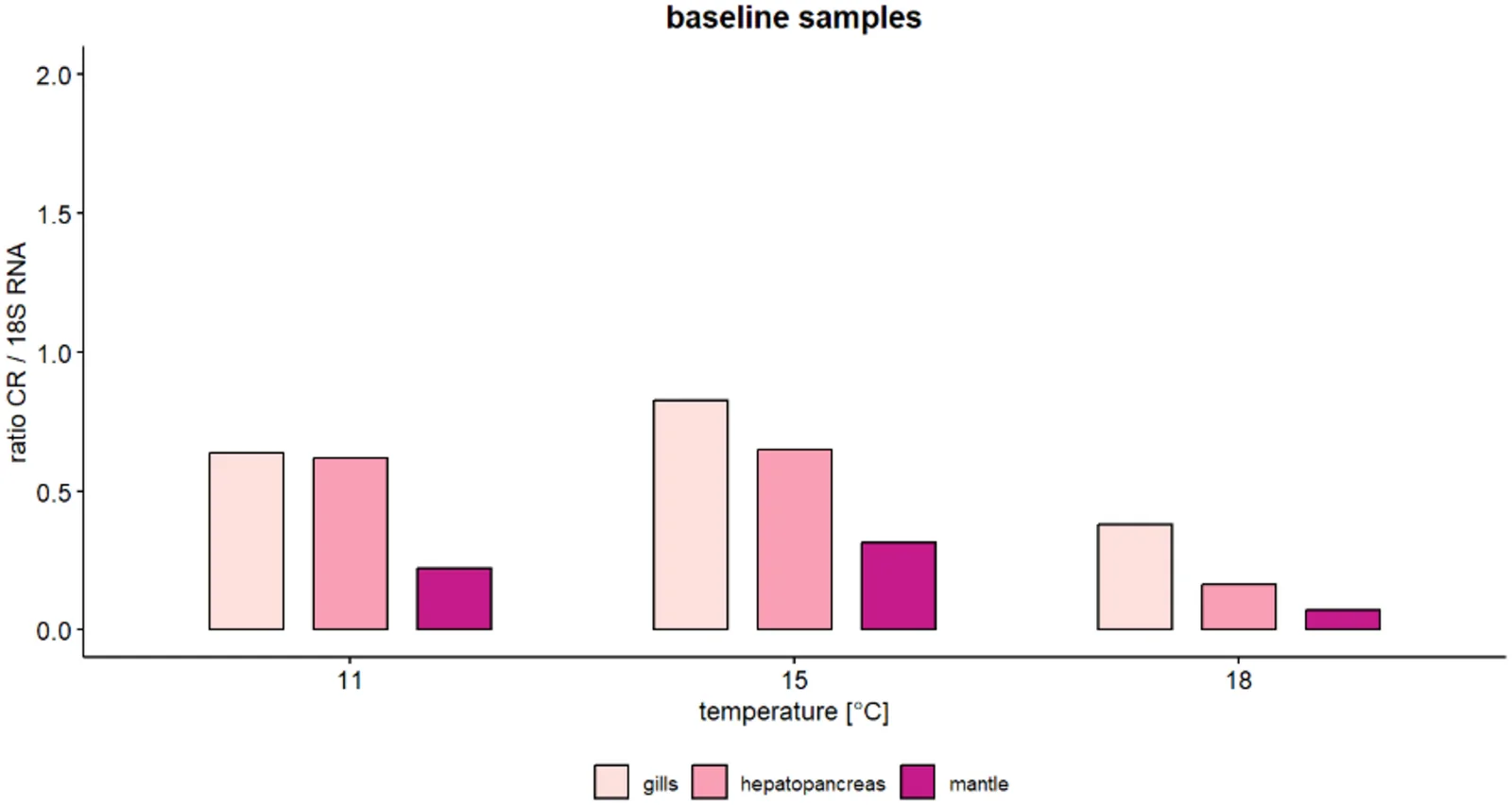
Relative gene expression of *CR* at different temperatures in gills, hepatopancreas and mantle tissue of *M. edulis* before exposure with TNT. Data are shown as the ratio of band intensity of *CR* to the band intensity of *18S rRNA* after 21 days of acclimatisation to the different temperature conditions and prior to TNT exposure

In this study, gene expression of *CR* was examined in blue mussels after exposure to different TNT concentrations at different temperatures by using semiquantitative PCR followed by densitometric analysis. The *18S rRNA* gene served as housekeeping gene. Figure 2 shows an example of a gel electrophoresis from gill tissue samples. It could be shown that *CR* mRNA expression is influenced by water temperature as well as TNT concentration. Samples taken after acclimatisation to the respective temperatures but before exposition to TNT already show differences in relative gene expression of *CR*. At 18 °C, a lower gene expression could be observed than at 11 °C and 15 °C, respectively (Fig. 3).

**Fig. 4:**
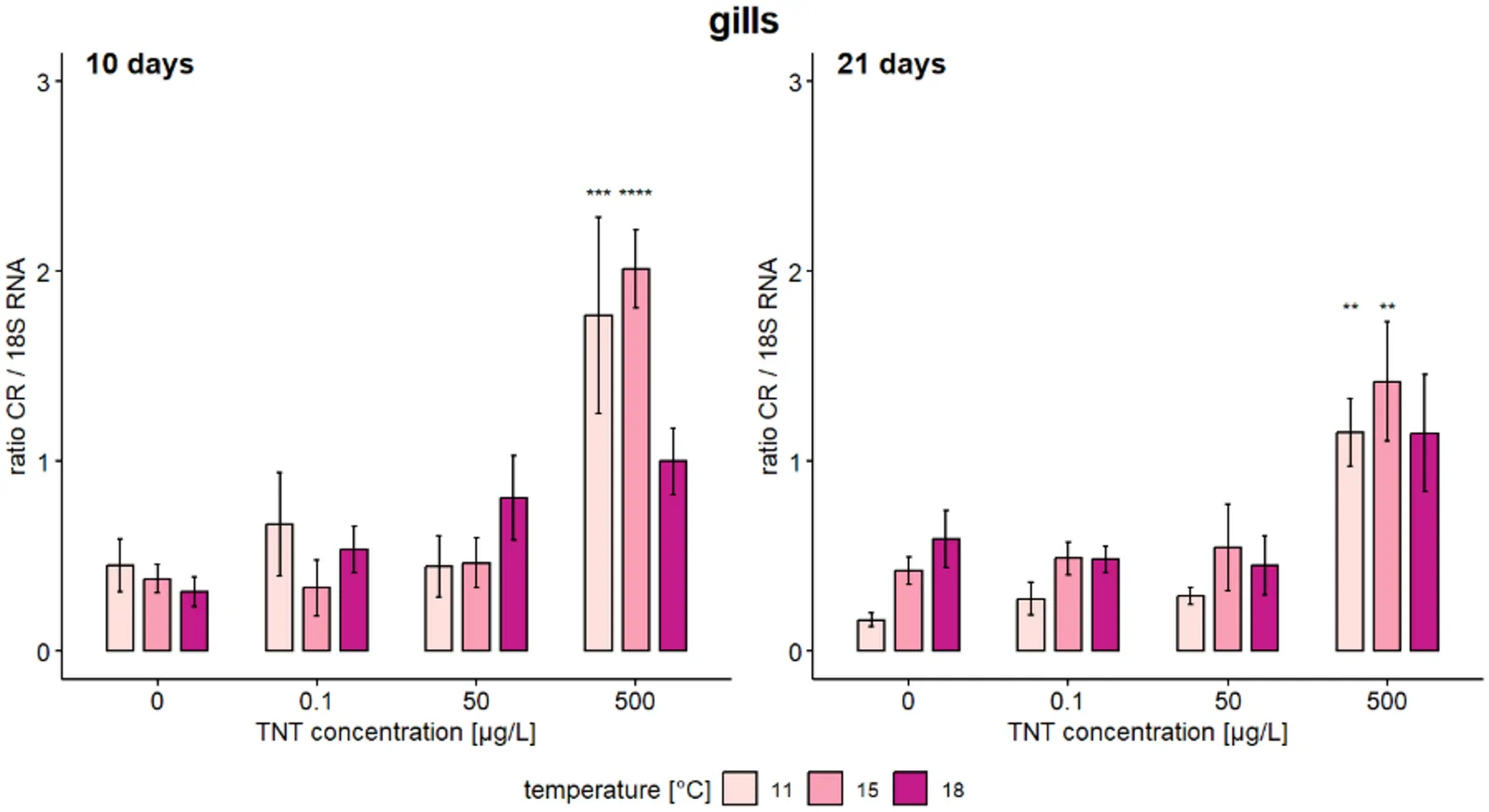
Relative gene expression of *CR* in the gills of *M. edulis* as a consequence of variations in exposure time and temperature as well as in TNT concentrations. Shown is the ratio of band intensity of *CR* to the band intensity of *18S rRNA* after 10 days (left) and 21 days (right), as well as of TNT exposure at different TNT concentrations and temperatures. Statistically significant changes compared to the within-temperature control group are indicated by **P < 0.01; ***P < 0.001; ****P < 0.0001

After exposure to 0.1 µg/L, 50 µg/L, and 500 µg/L TNT at 11 °C, 15 °C and 18 °C, respectively, changes in *CR* gene expression could be observed especially in gill tissue (Fig. 4). After 10 days of exposure, *CR* expression was significantly elevated in the 500 µg/L treatment group compared to the respective control groups in both the 11 °C and 15 °C treatment groups. For the 18 °C treatment group, elevated gene expression correlating with TNT concentration could also be observed, but lacks statistical significance. After 21 days, relative gene expression levels were generally lower, but still showed the pattern already visible after 10 days with significant elevation of gene expression for the treatment groups exposed to 500 µg/L TNT at 11 °C and 15 °C.

**Fig. 5:**
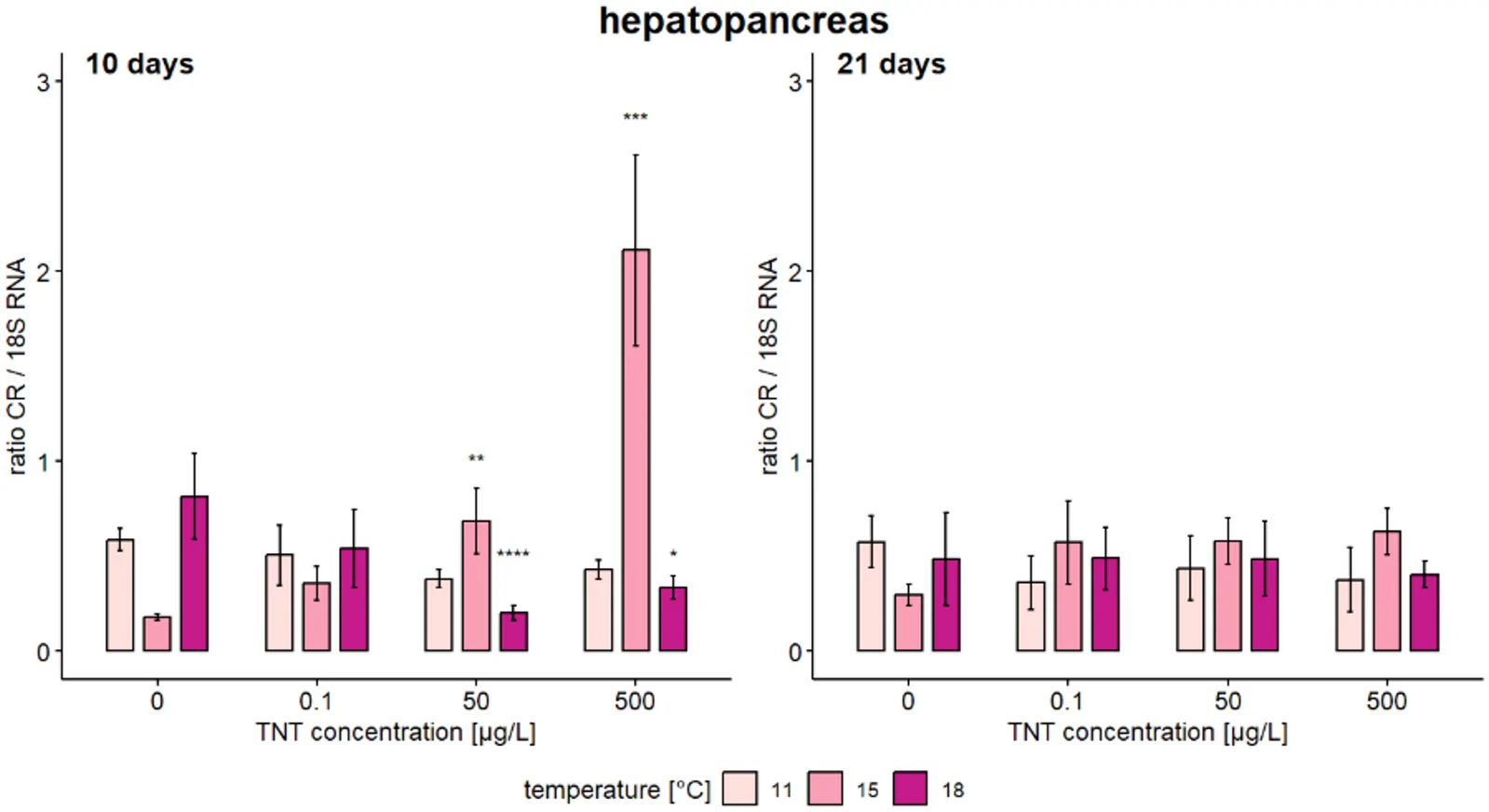
Relative gene expression of *CR* in the hepatopancreas tissue of *M. edulis* as a consequence of variations in exposure time and temperature as well as in TNT concentrations. Shown is the ratio of band intensity of *CR* to the band intensity of *18S rRNA* after 10 days (left) and 21 days (right), as well as of TNT exposure at different TNT concentrations and temperatures. Statistically significant changes compared to the within-temperature control group are indicated by **P < 0.01; ***P < 0.001; ****P < 0.0001

**Fig. 6:**
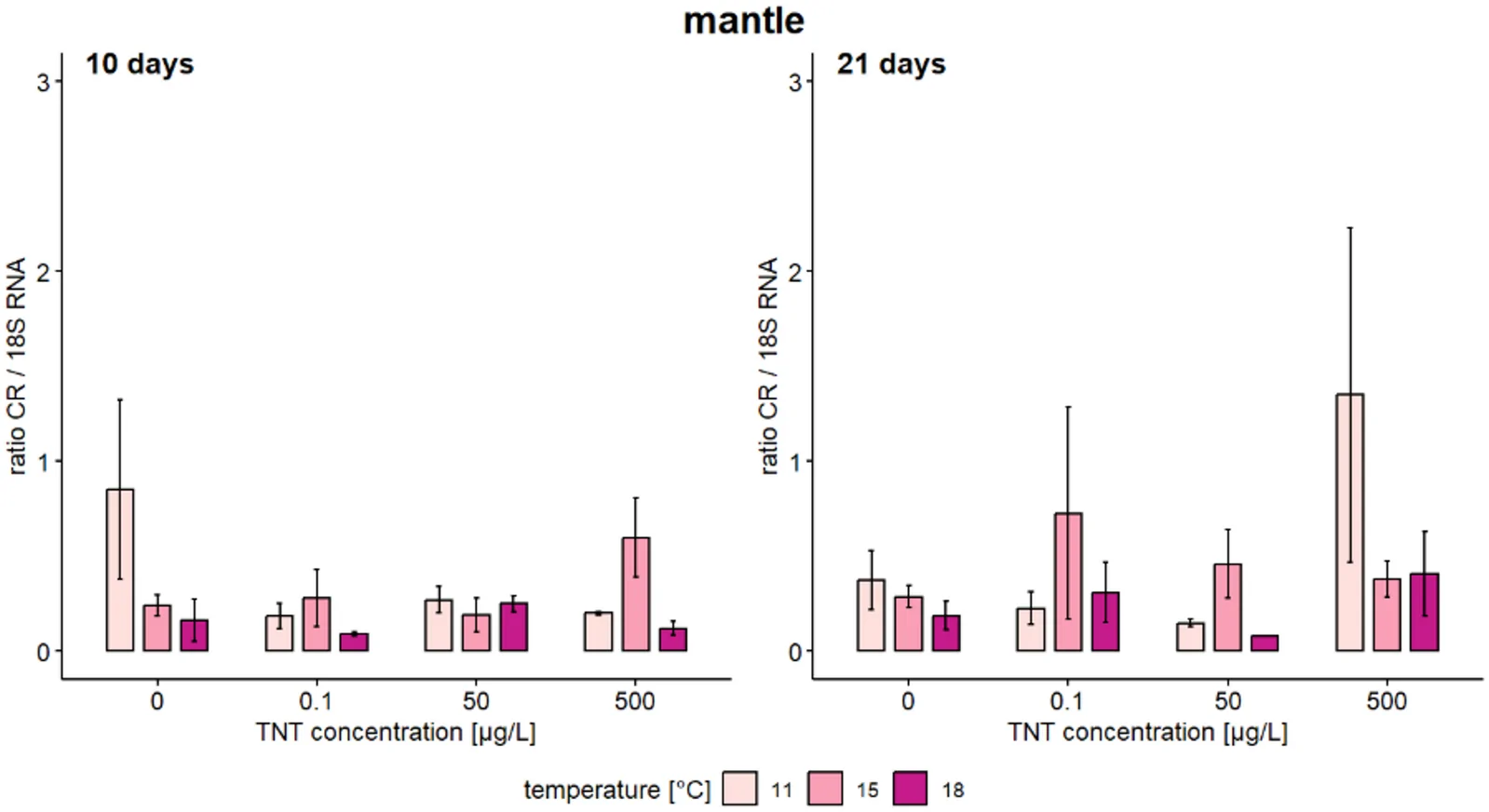
Relative gene expression of *CR* in the mantle tissue of *M. edulis* as a consequence of variations in exposure time and temperature as well as in TNT concentrations. Shown is the ratio of band intensity of *CR* to the band intensity of *18S rRNA* after 10 days (left) and 21 days (right), as well as of TNT exposure at different TNT concentrations and temperatures. Statistically significant changes compared to the within-temperature control group are indicated by **P < 0.01; ***P < 0.001; **** P < 0.0001

In hepatopancreas, after 10 days a significant decrease of gene expression compared to the control group could be observed for treatment groups exposed to 50 µg/L and 500 µg/L TNT at 18 °C, while for mussels exposed to the same concentrations of TNT at 15 °C a significant increase in gene expression could be seen (Fig. 5 left). After 21 days, no significant changes could be detected (Fig. 5 right). In mantle tissue, no significant TNT- or temperature-dependent changes of *CR* expression were observed (Fig. 6).

### Chemical analysis

**Fig. 7:**
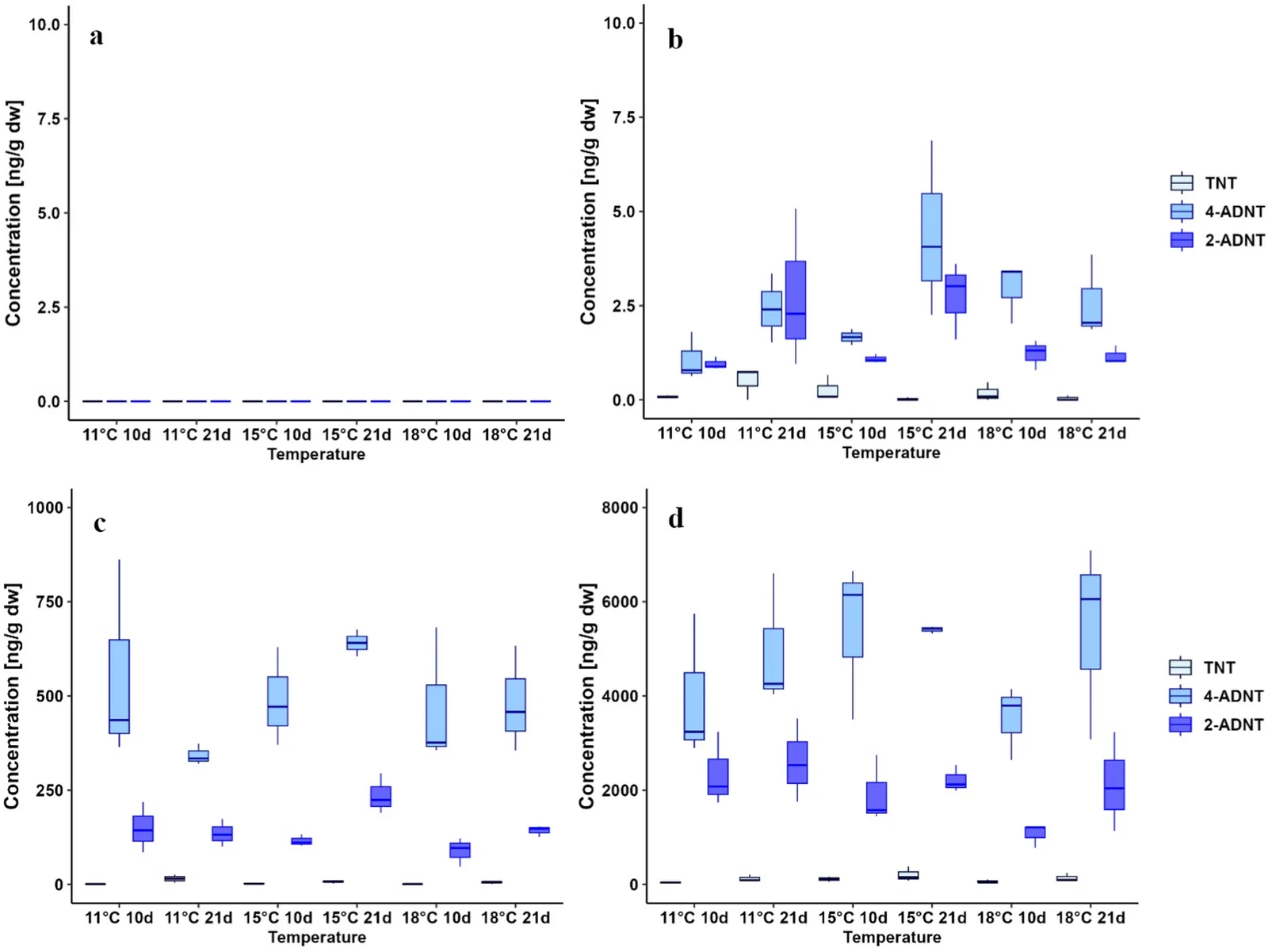
Tissue concentrations of TNT and its main metabolites 2-ADNT and 4-ADNT in freeze-dried whole tissue of the mussels exposed at temperatures of 11 °C, 15 °C, and 18 °C at concentrations of a 0 µg/L, b 0.1 µg/L, c 50 µg/L, and d 500 µg/L TNT in the water. No EC could be detected in the negative control samples. In mussels exposed to TNT, 4-ADNT could be found as the most abundant metabolite. TNT accounted for only between 0.5 and 8% of total EC found in the mussel tissue

The nominal water concentrations in the aquaria were confirmed using GC–MS/MS. No TNT or metabolites were found in the negative controls with regard to blue mussel and water samples from the respective aquaria. In the mussel samples, 4-ADNT was the most abundant TNT metabolite, while TNT was only found in a concentration range of 0.5–8% of the combined ADNTs. No trends were found between the three temperatures and two sampling times with regard to the concentration in the mussels from the tanks with the same water concentration (Fig. 7).

## Discussion

### Rising ocean temperatures increase pressure on marine ecosystems

Due to anthropogenic climate change, data project an increase of the global average ocean surface temperature of up to approximately 4 °C, assuming a very high emission scenario (Intergovernmental Panel on Climate Change (IPCC) 2023). Based on data already existing today, this is very likely to lead to significant changes of marine ecological niches and major challenges for the organisms inhabiting those. These organisms include, among others, blue mussels which are an integral part of marine ecosystems as well as marine food webs and, at the same time, serve as a sentinel organism in coastal pollution monitoring (Beyer et al. 2017; Strehse and Maser 2020). Studies could already show numerous effects of heat stress on different species among marine bivalves on behaviour, physiology as well as molecular immunity and antioxidative systems (reviewed in Masanja et al. 2023). Furthermore, there are also indications that exposure to environmental pollutants can impair bivalves’ ability to cope with heat stress (Lannig et al. 2006; Boutet et al. 2022). Evidence of the influence of heat stress on general gene expression levels could also already be shown in bivalves, even though a downregulation of gene expression could only be observed at higher temperatures than used in the present study for 22 genes involved in different metabolic pathways including energy metabolism, oxidative stress scavenging and cell cycle regulation in gill tissue of *Mytilus* spp. (Boutet et al. 2022). However, it should be considered that the combination of pollution and heat stress might cause downregulation of a broad variety of genes even at lower temperatures since similar synergisms could already be observed in bivalves exposed to other environmental pollutants such as cadmium (Lannig et al. 2006) and copper (Mlouka et al. 2019). Furthermore, there is evidence that exposure to chemical stressors negatively influences thermal tolerance of numerous aquatic organisms (Holmstrup et al. 2010). Since TNT and other EC leaking from corroding munitions are emerging to be one major pollutant of coastal marine habitats (Strehse et al. 2017; Appel et al. 2018; Beck et al. 2019; Esposito et al. 2023; Bünning et al. 2025), studies on the effects of those two combined stressors are vital to assess their impact on crucial species in coastal ecosystems.

### TNT causes adverse effects on different functional levels

It is well known that TNT is able to induce oxidative stress (reviewed in Adomako-Bonsu et al. 2024). Oxidative stress and ROS are able to induce cellular damages, e.g. due to the formation of toxic carbonyl adducts (Hoffmann and Maser 2007). Metabolic antioxidant systems play a crucial role in the mitigation of damages on genetic, cellular and functional levels caused by oxidative stress. Here, enzymes of the protein superfamily SDR such as CR are key protective mechanisms. This has been clearly demonstrated in humans as well as in numerous other species (Hoffmann and Maser 2007; Ebert et al. 2018). It has also been shown that TNT influences the gene expression of *CR* in *Daphnia magna* (Jacobsen et al. 2022) and blue mussels (Strehse et al. 2020)*.* However, studies are still pending to determine whether the increased mRNA production is converted into the corresponding protein to the same extent. Initial studies were conducted by Strehse (2020) using the blue mussel (*Mytilus* spp.). An increase in the activity of antioxidant defence systems in an organism is to be considered a warning indicator. It is already known that bivalves can be adversely affected by TNT (Rosen and Lotufo 2007a; Schuster et al. 2021, 2026). On the one hand, adverse effects can be observed at the level of single individuals. This includes a deterioration in general health, for example due to a shift in the energy status caused by increased stress and reduced production of byssus threads (Rosen and Lotufo 2007a). On the other hand, the effects of TNT contamination may even result in adverse effects on population dynamics by impairing spawning activity, embryonic development, and the survival of adult and juvenile mussels (Rosen and Lotufo 2007a).

### *CR* gene expression is influenced by TNT in the water as well as by temperature

Previous studies could already show that increased gene expression of *CR* in *Mytilus* spp. correlates with TNT concentrations in the water. Strehse et al. (2020) exposed mussels to TNT concentrations between 0.31 and 10 mg/L as well as in a munitions dumping area in the western Baltic Sea where EC concentrations between 3.3 µg/L and 3100 µg/L were measured (Beck et al. 2019). In both experiments, significantly increased mRNA expression levels of *CR* could be observed. Thus, concentrations between 0.1 µg/L and 500 µg/L were used at 11 °C, 15 °C, and 18 °C in the present study while in the lab study conducted by Strehse et al. (2020), mussels were exposed to TNT at 3 °C (96 h) and 9 °C (21 days).

Semiquantitative PCR analysis carried out in the present study showed that *CR* mRNA expression levels are indeed influenced by lower concentrations of TNT. Especially in gill tissue, a linear correlation between TNT concentration and relative *CR* mRNA expression could be shown similar to the results of the study conducted by Strehse et al. (2020). As gills are the first tissue to come into contact with pollutants in a filtrating organism like the blue mussel, it is obvious that they respond most strongly to an exogenous stressor such as TNT. Notably, expression patterns were observable after 10 days as well as after 21 days of exposure. In hepatopancreas, *CR* mRNA expression was shown to be influenced by exposition to TNT as well, but an effect was only detectable for higher concentrations upwards from 50 µg/L. In the laboratory study conducted by Strehse et al. (2020), statistically significant differences of *CR* mRNA expression in hepatopancreas tissue compared to the control group were only found at higher concentrations than tested here. Although the digestive gland is the most important metabolic organ in mussels (Lobo-da-Cunha 2019), these results indicate that *CR* expression in hepatopancreas tissue is not initially increased at TNT concentrations below 50 µg/L.

Furthermore, a potential influence of temperature on *CR* mRNA expression has to be considered since the water temperatures used in the present study were higher than those used in the laboratory exposure experiment conducted by Strehse et al. (2020). As an additional stressor in marine ecosystems, they should be addressed in combination with already existing as well as emerging chemical pollutants. First of all, the present study shows a lowered mRNA expression rate of *CR* in all tissues at 18 °C in the baseline samples compared to the two lower temperatures tested. In gill tissue, *CR* mRNA expression at 18 °C was lower than at the other temperatures in the mussels exposed to 500 µg/L TNT both after 10 and 21 days. In hepatopancreas, a lower expression rate of *CR* at 18 °C compared to lower temperatures was visible after 10 days in mussels exposed to 50 µg/L and 500 µg/L, respectively.

A review of existing studies clearly shows that synergisms between anthropogenic chemical pollutants and heat stress are very common (reviewed in Holmstrup et al. 2010). These findings have to be taken into account for assessment of biomarker data, because in this context the lack of gene expression upregulation should not automatically be interpreted as a sign of lower levels of oxidative stress or ROS as it also may indicate that the antioxidant systems are no longer functioning properly under the pressure of two combined stressors.

## Conclusion

In summary, this study adds vital information to further understand how blue mussels, as an integral part of many coastal marine ecosystems, are influenced by the combination of two emerging threats to marine life. Our data suggest a higher vulnerability to xenobiotic-induced oxidative stress in case of simultaneous presence of pollution from munitions and elevated water temperature. This should be taken into consideration when conducting further studies on the issue of climate change and simultaneous increase of dissolved TNT in the water. It is clear that not only the elevated water temperature itself, but also a higher dissolution of TNT may synergistically enhance the overall stress level affecting mussels and other marine biota in the future. It is therefore important to have molecular biomarkers combined with chemical analyses, which can both be used simultaneously to monitor the state of mussel health and concurrently be used to infer the environmental status of marine waters in respective future monitoring programmes.

## Data Availability

The biological data presented in this study will be published open access at PANGAEA data base.
